# Identification of *Saccharomyces cerevisiae* Genes Whose Deletion Causes Synthetic Effects in Cells with Reduced Levels of the Nuclear Pif1 DNA Helicase

**DOI:** 10.1534/g3.115.021139

**Published:** 2015-10-15

**Authors:** Jennifer L. Stundon, Virginia A. Zakian

**Affiliations:** * Department of Molecular Biology, Princeton University, New Jersey 08544

**Keywords:** Pif1, helicase, synthetic lethality, yeast, *S. cerevisiae*

## Abstract

The multifunctional *Saccharomyces cerevisiae* Pif1 DNA helicase affects the maintenance of telomeric, ribosomal, and mitochondrial DNAs, suppresses DNA damage at G-quadruplex motifs, influences the processing of Okazaki fragments, and promotes breakage induced replication. All of these functions require the ATPase/helicase activity of the protein. Owing to Pif1’s critical role in the maintenance of mitochondrial DNA, *pif1*Δ strains quickly generate respiratory deficient cells and hence grow very slowly. This slow growth makes it difficult to carry out genome-wide synthetic genetic analysis in this background. Here, we used a partial loss of function allele of *PIF1*, *pif1-m2*, which is mitochondrial proficient but has reduced abundance of nuclear Pif1. Although *pif1-m2* is not a null allele, *pif1-m2* cells exhibit defects in telomere maintenance, reduced suppression of damage at G-quadruplex motifs and defects in breakage induced replication. We performed a synthetic screen to identify nonessential genes with a synthetic sick or lethal relationship in cells with low abundance of nuclear Pif1. This study identified eleven genes that were synthetic lethal (*APM1*, *ARG80*, *CDH1*, *GCR1*, *GTO3*, *PRK1*, *RAD10*, *SKT5*, *SOP4*, *UMP1*, and *YCK1*) and three genes that were synthetic sick (*DEF1*, *YIP4*, and *HOM3*) with *pif1-m2*.

## Introduction

Pif1 family DNA helicases are found in all three kingdoms (reviewed in Bochman *et al.* 2010) . The best studied of these helicases is the founding member of the family, the *Saccharomyces cerevisiae* Pif1. There are two isoforms of Pif1 that depend on whether the first or second methionine is used to initiate translation of the mRNA (Schulz and Zakian 1994; Zhou *et al.* 2000). One isoform is targeted to the mitochondria (mt) where it is critical for the maintenance of mtDNA and for respiratory competence. The second isoform is localized to the nucleus and functions in multiple pathways that affect genome integrity. Pif1 is a negative regulator of telomere lengthening and *de novo* telomere addition by virtue of its ability to displace telomerase from DNA ends (Schulz and Zakian 1994; Boule *et al.* 2005; Phillips *et al.* 2015). It is required to generate long flap Okazaki fragments (Pike *et al.* 2009) and to promote breakage induced replication (Saini *et al.* 2013; Wilson *et al.* 2013). Pif1 promotes DNA replication through G-quadruplex (G4) motifs, which are sequences that form G4 structures *in vitro*, and suppresses DNA damage at G4 motifs (Ribeyre *et al.* 2009; Paeschke *et al.* 2011, 2013; Piazza *et al.* 2012). Additionally, Pif1 helps maintain the replication fork barrier (RFB) in the ribosomal DNA (rDNA) (Ivessa *et al.* 2002). Although Pif1 has weak unwinding activity on conventional 5′ tailed duplex DNA substrates, it robustly unwinds G4 structures and RNA/DNA hybrids *in vitro* (Boule and Zakian 2007; Ribeyre *et al.* 2009; Paeschke *et al.* 2011; Zhou *et al.* 2014).

Despite its multiple and diverse functions, *PIF1* is not an essential gene. We anticipated that other genes might act in parallel with *PIF1* to carry out its various cellular functions. *S. cerevisiae* encodes a second Pif1 family helicase, Rrm3, whose helicase domain is 40% identical to that of Pif1. However, the functions of Rrm3 and Pif1 are largely nonoverlapping, except at G4 motifs (Paeschke *et al.* 2013). Rrm3 does not appear to be a backup for Pif1 at many of its genomic targets (Ivessa *et al.* 2000, 2002; O’Rourke *et al.* 2005). We predicted that *PIF1* might have synthetic interactions with genes involved in regulating telomere length, Okazaki fragment maturation, breakage induced replication, and G-quadruplex unwinding. Additionally, because Pif1 binds *in vivo* to the promoters of ∼130 genes (C. F. Chen, S. Pott, and V. A. Zakian, unpublished results), Pif1 might have as yet undescribed roles in transcriptional regulation, which could result in interactions with transcription factors. We anticipated that we might detect indirect synthetic lethal relationships owing to Pif1’s effect on gene expression and/or genome integrity. In addition, as *pif1-m2* cells are more sensitive to proteasomal inhibition and have a higher basal level of autophagy, *pif1-m2* cells may be more dependent on the proteasome for cellular maintenance and survival (J. L. Stundon and V. A. Zakian, unpublished results). Thus, *pif1-m2* might have synthetic interactions with other genes with roles in autophagy and proteasomal function.

### Rationale for screen

As we are particularly interested in the nuclear functions of Pif1, we sought to identify genes whose deletion affected the viability of or reduced the growth rate of *pif1-m2* cells, which are deficient in the nuclear form of Pif1 (Schulz and Zakian 1994; Zhou *et al.* 2000). This strategy avoided the difficulty of using *pif1*Δ cells, which are very slow growing, behavior that might obscure synthetic interactions.

## Materials and Methods

### Screen design

**Figure 1:**
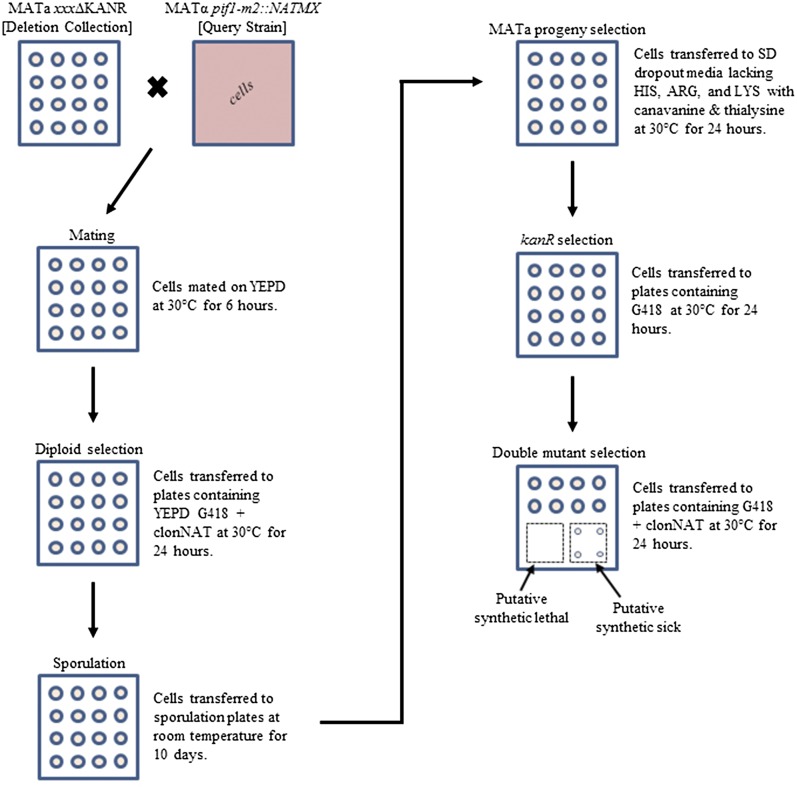
Schematic of steps for synthetic genetic analysis. All cells grown in quadruplicate as shown. Use of the *ho*Δ*NATMX* control strain in parallel is not pictured. YEPD, yeast extract peptone dextrose.

Strains and plasmids used in this study are listed in Table 1 and Table 2. The prototroph deletion collection, which contains 4783 strains with a single deletion of a nonessential gene, tagged with the *kanMX* antibiotic resistance marker, was used. The *pif1-m2* query strain was created using the pvs31 plasmid, an integrating plasmid with a *URA3* selectable marker (Schulz and Zakian 1994). The pvs31 plasmid was linearized with *Hin*dIII (NEB) and transformed into DBY11087 using lithium acetate transformation (Becker and Lundblad 2001). After introduction of the *pif1-m2* mutation, the *natMX* resistance cassette was added proximal to the *pif1-m2* gene (Goldstein and McCusker 1999). The *pif1-m2* mutation was confirmed by polymerase chain reaction and sequencing and shown to segregate 2:2 with the *natMX* marker. Mating, sporulation and selection were performed as described (Tong and Boone 2006). Synthetic genetic analysis was performed as described (Tong and Boone 2006) as outlined in Figure 1. Briefly, the mutant and query strains were grown on yeast extract peptone dextrose media at 30°, and then mated, and diploids were selected using yeast extract peptone dextrose with G418 + clonNAT. The strains were transferred to sporulation media, then MATa haploids were selected using drop out media lacking HIS, ARG and LYS with canavanine and thialysine, followed by selection with drop out media with G418, and finally by selection of double mutant haploids on drop out media with G418 + clonNAT.

**Table 1:** Strains used in this study

Strain Name	Mutation, Strain Background, Previous Name/Previous Study if Applicable
Query-*pif1m2*::*NATMX*	*pif1m2* mutation and NATMX cassette added to*:* DBY11087; S288C, MATα, *his3*∆ *leu2*∆ *ura3*∆ *lyp1*∆ *met15*∆*cyh2*∆ *LYS2 can1*::*Pste2-S.P. his5*
Control-*ho*∆::*NATMX*	*ho*∆::*natMX* deletion added to: DBY11087; MATα, *his3*∆ *leu2*∆ *ura3*∆ *lyp1*∆ *met15*∆*cyh2*∆ *LYS2 can1*::*Pste2-S.P. his5*
Prototrophic Deletion Mutation Array	DBY15001 W303 derived, MATa. Prototrophic deletion collection; created by Drs. Amy Caudy and David Hess. (Klosinska *et al.* 2011)

**Table 2:** Plasmids used in this study

Plasmid Name	Description of Plasmid Use
pAG25	Insertion of the NATMX cassette (Goldstein and McCusker 1999)
pvs31	Insertion of the *pif1m2* mutant via pop-in/pop-out (Schulz and Zakian 1994)

### Phenotypes

Each strain was mated in quadruplicate with the control (*ho*Δ::*NATMX)* and query (*pif1-m2*::*NATMX)* strains to generate double mutant diploids, which were then sporulated. Double mutant haploid clones (*i.e.*, *pif1-m2 geneX*Δ) were derived from these spores. Strains that failed to form viable double mutant haploids when mated with the *pif1-m2* strain but successfully formed viable double mutant haploids when mated with the control strain were considered putative synthetic lethal interactors. Strains that formed slow growing double mutant haploids when mated with the *pif1-m2* strain (determined by visual inspection as being <50% of the size of either single mutant) were considered candidates for putative synthetic sick interactors. The use of the robotic pins, and the mixing steps utilized in the RoTOR robot (Singer, RoTOR-HDA), prevented the visualization of less severe synthetic growth differences. The synthetic sick mutants were not tested for mitochondrial proficiency.

### Verification of mutants

Each putative synthetic relationship was re-examined by mating the appropriate strains by hand, selecting for diploids, which were sporulated and the resulting tetrads dissected. In some cases, random spore analysis was used as described (Lichten 2014).

## Results

The genetic screen identified eleven genes that were synthetic lethal and three genes that were synthetic sick with *pif1-m2*. Surprisingly, this screen did not identify any of the over 100 genes that have been shown or proposed to encode a helicase as having a synthetic relationship with *pif1-m2*, including *sgs1*Δ and *rad3*Δ, which were shown previously to be synthetic sick with *pif1*Δ (Wagner *et al.* 2006; Moriel-Carretero and Aguilera 2010). The fact that *pif1-m2* is not a null allele and retains residual nuclear function (Schulz and Zakian 1994) most likely explains why we did not observe synthetic relationships between *pif1-m2* and other helicases and/or genes previously reported to have a synthetic phenotype with *pif1*Δ. Alternatively, synthetic phenotypes reported earlier may be due to the respiratory deficiencies, rather than the nuclear defects, of *pif1*Δ cells (Supporting Information, Table S1). It is also possible that the W303 based prototrophic deletion collection used here may contribute to the differences between this study and earlier analyses, as several earlier studies were completed in the BY4741 background (Pan *et al.* 2004, 2006), others used S288c (Zhang and Durocher 2010), and some used a combination of strain backgrounds (Osman *et al.* 2009; Moriel-Carretero and Aguilera 2010).

### Synthetic lethal genes

Eleven genes were identified whose deletion from a *pif1-m2* cell generated inviable cells. Here, we list those genes and provide some information on their functions and potential interactions with *PIF1*.

*APM1* encodes a protein that is a subunit of the clathrin-associated protein complex. It is involved in the vesicular transport process (Nakayama *et al.* 1991; Stepp *et al.* 1995). *apm1*Δ cells have abnormal vacuolar transportation and abnormal Golgi protein sorting (Phelan *et al.* 2006).

*ARG80* encodes a transcription factor that is involved in the regulation of arginine responsive genes (Dubois *et al.* 1987). *arg80*Δ cells have abnormal vacuolar morphology and decreased fitness (Michaillat and Mayer 2013). Given that Pif1 binds promoters of many genes (see *Introduction*), this synthetic phenotype may reflect a transcriptional problem in the double mutant.

*CDH1* encodes a protein that activates the anaphase promoting complex/cyclosome (Visintin *et al.* 1997; Harper *et al.* 2002; Woodbury and Morgan 2007). Cdh1 is a cell-cycle regulated protein that directs the ubiquitination of cyclins and helps to orchestrate the mitotic exit from the cell cycle. *cdh1*Δ cells have increased telomere length and abnormal cell cycle progression (Visintin *et al.* 1997; Askree *et al.* 2004). Pif1 abundance is also cell-cycle regulated in a proteasome dependent manner, suggesting a potential relationship between Cdh1 and Pif1 (Mateyak and Zakian 2006). In addition, the essential telomerase subunit Est1 is cell cycle regulated (Taggart *et al.* 2002) in a proteasome and Cdh1-dependent manner (Osterhage *et al.* 2006; Ferguson *et al.* 2013). Moreover, many of the proteins that copurify with yeast telomerase, as determined by mass spectrometry analysis, affect ubiquitin and proteolysis (Lin *et al.* 2015). Thus, the lethality of *pif1-m2 cdh1*Δ cells may be due to impaired proteolysis that affects telomere length or other Pif1 functions.

*GCR1* encodes a DNA binding protein that interacts with the transcriptional activator Gcr2 to promote transcriptional activation of genes involved in glycolysis (Clifton *et al.* 1978; Holland *et al.* 1987). As with *ARG80*, this interaction may be due to a transcriptional function of Pif1.

*GTO3* encodes a glutathione transferase with a poorly defined function. It is putatively localized to the cytosol and *gto3*Δ cells have abnormal vacuolar morphology (Herrero 2005; Garcera *et al.* 2006).

*PRK1* encodes a serine/threonine protein kinase that is involved in cytoskeletal organization and actin function (Byrne and Wolfe 2005; Zeng and Kinsella 2010). Endocytosis is reduced in *prk1*Δ cells (Henry *et al.* 2003).

*RAD10* encodes a single-stranded DNA endonuclease with roles in both nucleotide excision repair and single-strand annealing-mediated recombination (Ivanov and Haber 1995; de Laat *et al.* 1999; Symington 2002). Pif1 inhibits telomerase-mediated double-strand break repair (Schulz and Zakian 1994). Rad10 promotes the creation of gross chromosomal rearrangements (GCR), which are increased in both *pif1-m2* and *pif1*Δ cells (Myung *et al.* 2001; Hwang *et al.* 2005; Piazza *et al.* 2012; Paeschke *et al.* 2013). We speculate that *pif1-m2 rad10*Δ cells may be inviable due to combined defects in two different DNA repair pathways. Surprisingly, even though Rad1 and Rad10 act together in nucleotide excision repair and single strand annealing, this screen did not identify a synthetic relationship between *pif1-m2* and *RAD1*. This result might indicate that *RAD10* has a function that is distinct from *RAD1*, which is responsible for the synthetic relationship between *RAD10* and *pif1-m2*. For example, a telomere-dedicated single strand annealing pathway that is *RAD10*- but not *RAD1*-dependent might act on the highly repetitive telomeric DNA.

*SOP4* encodes an endoplasmic reticulum membrane protein that is involved in the export of Pma1 and Pma1-7, proteins that regulate cytoplasmic pH and help to maintain the plasma membrane potential from the endoplasmic reticulum (Luo and Chang 1997; Luo *et al.* 2002). *sop4*Δ cells have abnormal vacuolar morphology (Michaillat and Mayer 2013).

*SKT5* encodes a protein that activates the chitin synthetase Chs3 that helps form spore walls (Iwamoto *et al.* 2005). *skt5*Δ cells have decreased vegetative growth rate and decreased viability (Kozubowski *et al.* 2003; Byrne and Wolfe 2005).

*UMP1* encodes a protein that is a chaperone required for the maturation of the 20S proteasome (Ramos *et al.* 1998; Ishikawa *et al.* 2005). In *ump1*Δ cells, the proteasome is functionally impaired (Ramos *et al.* 1998) and data from our lab shows that *pif1-m2* cells are more sensitive to proteasomal inhibition (J. L. Stundon and V. A. Zakian, unpublished results). *ump1*Δ cells with decreased nuclear Pif1 may be inviable due to strain on the proteasomal machinery. As with *CDH1*, the synthetic effects of *pif1-m2* and *ump1*Δ may result from impaired Pif1 proteolysis.

*YCK1* encodes a palmitoylated membrane-bound casein kinase that is involved in endocytic trafficking and glucose sensing (Robinson *et al.* 1992; Reddi and Culotta 2013). *yck1*Δ cells have abnormal vacuolar morphology (Michaillat and Mayer 2013).

### Synthetic sick genes

Our screen also identified genes whose deletion in a *pif1-m2* cell resulted in a slow growth phenotype where double mutants grew to <50% of the size of the single mutant strain. This analysis identified three such genes, *DEF1*, *YIP4*, and *HOM3*.

The identification of a synthetic relationship between *PIF1* and *DEF1* was particularly exciting, as both genes function in genome maintenance, telomere length, and maintenance of mtDNA. Def1 forms a complex with Rad26, a protein that functions in transcription-coupled repair (Woudstra *et al.* 2002; Somesh *et al.* 2005; Jordan *et al.* 2007). Def1 also plays a role in the maintenance of telomeres, as *def1*Δ telomeres are 200 bp shorter than the wild-type length of ∼300 bp (Chen *et al.* 2005). Like *pif1*Δ cells, *def1*Δ cells display increased mitophagy and abnormal vacuolar morphology (Michaillat and Mayer 2013; Bockler and Westermann 2014). We speculate that the reduced growth rate of the *pif1-m2 def1*Δ strain is due to their shared roles in DNA repair and telomere length.

*YIP4* encodes a protein that interacts with Rab GTPases and is involved in vesicle-mediated transport (Samanta and Liang 2003; Inadome *et al.* 2007).

*HOM3* encodes an aspartate kinase that is localized to the cytoplasm and catalyzes methionine and threonine biosynthesis (Mountain *et al.* 1991).

## Discussion

The identified genes, whose deletion had synthetic effects with *pif1-m2*, support the known multifunctional nature of Pif1. In addition to its multiple described functions, our data suggest potential new roles for Pif1 in proteasome function, transcription coupled repair, endocytosis and vacuolar morphology, although some of these effects may be indirect if Pif1 has a transcriptional function. Future work will focus on elucidating the connections between *PIF1* and the genes identified in this study, with a particular interest in examining the relationship between *PIF1*, *CDH1*, *RAD10*, and *DEF1*, as these genes all affect telomeres and/or DNA repair (Schulz and Zakian 1994; Woudstra *et al.* 2002; Askree *et al.* 2004; Chen *et al.* 2005). A second major focus will be on the role of Pif1 in proteasomal function as the synthetic relationships between *pif1-m2* and *CDH1* and *UMP1* reported here, coupled with unpublished data from our lab, suggest a role for Pif1 in proteasomal function, which may be important for cells to tolerate stress. The synthetic effects of *CDH1* and *pif1-m2* and *UMP1* might additionally help elucidate a role for *PIF1* in autophagy. Future work will also examine the role of *PIF1* on transcriptional regulation, which may help identify indirect interactions that are responsible for some of the synthetic genetic relationships reported here.

## Supplementary Material

Supporting Information
