# Supplementary material for: Identification of Saccharomyces cerevisiae Genes Whose Deletion Causes Synthetic Effects in Cells with Reduced Levels of the Nuclear Pif1 DNA Helicase
Source: G3 (Bethesda). 2015 Oct 15;5(12):2913–8. doi: 10.1534/g3.115.021139 (PMC4683662; doi:10.1534/g3.115.021139)
Supplement: Supporting Information [file supp_5_12_2913__index.html]

Identification of Saccharomyces cerevisiae Genes Whose Deletion Causes Synthetic Effects in Cells with Reduced Levels of the Nuclear Pif1 DNA Helicase — Supporting Information 

# Identification of *Saccharomyces cerevisiae* Genes Whose Deletion Causes Synthetic Effects in Cells with Reduced Levels of the Nuclear Pif1 DNA Helicase

## Supporting Information for Stundon and Zakian, 2015

**Files in this Data Supplement:**

- Table S1 - Previously reported synthetic effects with pif1Δ. (.pdf, 127 KB)
