# Supplementary material for: Identification of Saccharomyces cerevisiae Genes Whose Deletion Causes Synthetic Effects in Cells with Reduced Levels of the Nuclear Pif1 DNA Helicase
Source: G3 (Bethesda). 2015 Oct 15;5(12):2913–8. doi: 10.1534/g3.115.021139 (PMC4683662; doi:10.1534/g3.115.021139)
Supplement: Supporting Information [file supp_g3.115.021139_TableS1.pdf]

Table S1. Previously reported synthetic effects with *pif1Δ*

Gene	Synthetic Effect	Experiment Type
TOP3	Lethal	Manually Curated(WAGNER *et al.* 2006)
CCR4	Growth Defect	High Throughput(PAN *et al.* 2006)*
DIA2	Growth Defect	Manually Curated(BLAKE *et al.* 2006) & High Throughput(PAN *et al.* 2006)*
EAF3	Growth Defect	High Throughput(LIN *et al.* 2008)*
EAF6	Growth Defect	High Throughput(LIN *et al.* 2008)*
LTE1	Growth Defect	High Throughput(YE *et al.* 2005)*
MMS22	Growth Defect	High Throughput(PAN *et al.* 2006)*
PHB1	Growth Defect	High Throughput(OSMAN *et al.* 2009)
POP2	Growth Defect	High Throughput(PAN *et al.* 2006)*
RAD27	Growth Defect	High Throughput(PAN *et al.* 2006)*
RAD3	Growth Defect	Manually Curated(MORIEL-CARRETERO and AGUILERA 2010)
RAD53	Growth Defect	High Throughput(PAN *et al.* 2006)*
RRD1	Growth Defect	Manually Curated(ZHANG and DUROCHER 2010)
SGS1	Growth Defect	High Throughput(PAN *et al.* 2004) & Manually Curated(WAGNER *et al.* 2006)
TLC1	Growth Defect	Manually Curated(DEWAR and LYDALL 2010)
YKU70	Growth Defect	Manually Curated(ZHANG and DUROCHER 2010)

*Indicates that a hit from a high throughput screen was verified through random spore analysis, tetrad dissection or both.
